# An Historical Account of the Statistics and the Moral Condition of Foundlings; to Which Are Added a Hundred Tables

**Published:** 1842-04

**Authors:** 


BRITISH AND FOREIGN

### MEDICAL REVIEW,

FOR APRIL, 1842.

### PART FIRST.

### Analytical and Critical Reviews.

### Art. I.

1. *Histoire Statistique et Morale des Enfans Trouvés, suivie de cent Tableaux.* Par J. F. Terme et J. B. Monfalcon.—*Paris et Lyon*, 1837. 8vo, pp. 504.

*An Historical Account of the Statistics and the Moral Condition of Foundlings; to which are added a Hundred Tables.* By J. F. Terme and J. B. Monfalcon.—*Paris and Lyons*, 1837.

2. *Nouvelles Considérations sur les Enfans Trouvés.* Par J. F. Terme et J. B. Monfalcon.—*Lyon*, 1838. pp. cviii.

*Further Considerations upon Foundlings.* By J. F. Terme and J. B. Monfalcon.—*Lyons*, 1838.

3. *Des Hospices des Enfans Trouvés en Europe et principalement en France, etc.* Par B. B. Remacle.—*Paris*, 1838. 8vo, pp. 405.

*On Foundling Hospitals in Europe and principally in France, from their origin to the present day.* By B. B. Remacle.—*Paris*, 1838. *With a quarto volume of Official Statistical Documents.*

4. *Recherches sur les Enfans Trouvés, les Enfans Naturels, et les Orphelins en France, etc.* Par L'Abbé A. H. Gaillard.—*Paris*, 1837. 8vo, pp. 400.

*Researches into the Statistics and the Moral Condition of Foundlings, Illegitimate Children, and Orphans, and the laws respecting them in France, and in many other countries of Europe.* By the Abbé A. H. Gaillard.—*Paris*, 1837.

The attention of our continental neighbours has recently been directed to the improvement of their laws for the relief and support of the poor. Among the subjects connected therewith, none has given rise to greater difference of opinion than that of the provisions to be made for deserted and illegitimate children, and the means to be employed in order to check, if possible, the yearly increase in their numbers. In order to solve this problem it was necessary to investigate many questions more or less nearly connected with our art, and all of which present great interest to the man of science. We therefore need make no apology for presenting to our readers a brief account of the Foundling system in France.

Our readers are doubtless aware that, in France, as in all Catholic countries, the wants of the needy are supplied from other sources, and by means quite different from those resorted to in most Protestant lands. Foundling hospitals, intended to provide for the support of deserted children, and to open to unmarried females an asylum where the child would be cared for and the mother's shame hidden, form an important feature in the system of Catholic charity. Public opinion in England has declared, rightly as we think, against these institutions; but still it was a noble thought to cover the sins and frailties of the weak or the wicked with the mantle of charity, and to obviate the consequences of those vices which religion was not strong enough to root out.

Magdalenism and foundling hospitals were the offspring of the middle ages; and at a time when the darkest ignorance prevailed, and Christianity was most corrupt, charity was busied with works such as were never dreamt of in the most enlightened days of Greece and Rome. The induction of abortion was almost universally practised at Rome; infanticide was extremely common, and desertion of children was practised by persons in all ranks of life. But there was no foundling hospital at Rome; and no one saved the lives of these poor infants, except wretches who after having blinded or mutilated them, sent them to beg in the streets, and lived on their earnings. And these things were done in the days of Augustus Cæsar, of Cicero and Virgil.

The early Christian emperors endeavoured to diminish the frequency of infanticide, but the sources of the evil lay too deep to be reached by the mere denouncement of punishments against the crime. The condition of foundlings, however, was somewhat improved, since they no longer became the property of any one who cared to rear them, while Justinian even founded institutions for their support. Of the exact nature of these *Brephotrophia* we are ignorant, but they continued to exist, and the laws of Justinian remained in force, until the fall of the Eastern empire.

In order to form a correct estimate of the charitable institutions of the middle ages we ought to bear in mind the condition of society at that time. Not only did the most profound ignorance pervade all ranks, but universal depravation of morals had reached a point of which it is almost impossible to form any conception. At the same time wretchedness and want were wide spread and extreme. Parents were driven by absolute destitution to destroy or abandon their new-born infants, or they sold their children into slavery to procure bread for themselves.* A cold, calculating charity, fearing to succour the wretched lest indiscriminate relief should minister to idleness, or which would withhold help from perishing infants lest mothers should forget their duty to their children, could in those days have done no good. Christianity inculcates mercy, it teaches us to clothe the naked and feed the hungry, while we are not so pure that we can be our brother's judges, or refuse to relieve distress because by so doing we may support the unworthy. Such was the creed†

* The moral state of society in the middle ages is fully displayed in Meiner's Historische Vergleichung der Sitten, &c. des Mittelalters.—Hannover, 1793. Band i. Abschn. iv.

† The influence of religion on the mind in the middle ages is very eloquently set forth by Cibrario, Della Economia Politica del Medio Evo. Lib. ii. cap. i.

which made the church in western Europe the protectress of foundlings. A marble vessel stood outside the church door where women were encouraged to place their children, and to those who would have died of hunger and want, shelter was thus given and a nurse to tend them. For this support indeed they paid the price of their liberty, becoming the property of the *nutricarii* who brought them up, or else the serfs of the church which had defrayed the expenses of their education. But neither the relief afforded by the church, nor the benefactions of charitable individuals, nor even the hospitals in one or two large towns, as at Milan and Sienna, were sufficient to meet the growing evil.*

The stirring times of the Crusades brought with them many benefits, among which the institution of the orders of Hospitallers† was by no means the least. Their duty was to entertain strangers and pilgrims, whom some of their body were bound to defend from danger, to visit and relieve the poor and the sick, or to receive them into the hospitals, and to provide for orphans and deserted children. None of these societies, however, devoted themselves to the care of foundlings so zealously as the brotherhood of the Holy Ghost, which consisted at the time of its foundation, like the other orders of Hospitallers, principally of lay members. Guy of Montpellier, who had founded an hospital in his native city, was appointed master of it, as well as of the hospital *Santo Spirito in Sassia*, at Rome, on the incorporation of the society in 1204, by Pope Innocent III. It was supported by voluntary contributions levied throughout Christendom, and at one time houses of the order existed in almost every country of Europe.‡ As abuses, however, crept into these institutions, they were either suppressed or at any rate entirely diverted from their original purpose, and the lot of the foundlings became in consequence much worse. The law of France indeed declared them to be free, but the regulations which it made for their maintenance were by no means effective, and after the overthrow of all charitable institutions during the wars of the League, the resources of the church were quite inadequate to their support. Foundlings in Paris were laid on a couch at the entrance to the cathedral of Notre Dame, and a priest stood by who cried to all that passed, "Bestow your charity on these poor foundlings."

This state of things continued till near the middle of the seventeenth century; and in the brilliant days of Cardinal Mazarin and Anne of Austria, new-born infants might be seen dead or dying in the streets, or taken out of the Seine by the police. Madame Legras, a benevolent widow, turned her house into a foundling hospital, and, assisted by her two servants, provided for as many as her means would allow. At her death she left money to endow the institution, but the persons who succeeded her in the charge of the hospice abused their trust, neglected the

* A very elaborate account of the hospitals and charitable institutions of the middle ages will be found in Krüger's Archiv für Waisen und Armenerziehung.—Hamburg, 1825-8. Unfortunately the work was never completed, and the historical notices do not come lower than the foundation of the hospital at Milan in 787.

† For an account of the Hospitallers before they degenerated into military corporations, see Ersch und Gruber's Allgemeine Encyklopädie der Wissenschaften und Künste. Art. Hospitalliter.

‡ The laws of this and of the other orders of Hospitallers, and a sketch of their history, will be found in Brockie's Codex Regularum Monasticarum et Canonicarum, &c. Augustæ Vindel, 1759. Tom. vi. folio. For the regulations of the order of the Holy Ghost, see tome v. p. 495.

children, and even sold them for the paltry sum of twenty sous. Curiosity led St. Vincent de Paul to visit the hospice; he was shocked at the sight, and having called together some charitable ladies, begged them to assist him in forming an hospital for foundlings. In 1638 they hired a small house for twelve children; but, meeting with the patronage of the king and queen, they were soon enabled to operate on a larger scale. It was not, however, until 1670, some years after the death of St. Vincent de Paul, that the Hospice des Enfans Trouvés was declared to be one of the hospitals of Paris. Its fame soon spread, children were sent to it from all parts of France, and similar institutions were formed in some large towns. Still no adequate provision was made for the support of foundlings throughout the country, and disputes, in which the infants were invariably the sufferers, frequently took place between the communes and the lords justices, as to the duty of providing for them.

The revolution of 1789 put an end to these disputes. All foundlings were now placed under the uniform protection of the law, and hospices were ordered to be opened throughout France for their reception. It was declared an honorable distinction to be the mother of a bastard child,* and the state made large promises of the care it would take for the education of these "*enfans de la patrie.*" The financial difficulties of the country, however, rendered the fulfilment of any of these promises impossible. The Sisters of Charity too had been driven away, and the interior of the hospices presented a scene of gross mismanagement and utter confusion. A better order of things was introduced when Napoleon ascended the throne. The Sisters of Charity were recalled in 1804, and the provisions for the support of foundlings were made the subject of an imperial decree in 1811. In most points this decree confirmed the enactments of the republic, but, by directing the establishment of a *tour* † at every hospice it introduced a system of secret and uncontrolled admissions, against which many of the provincial administrations remonstrated. Their remonstrances, however, were of no avail, since Napoleon wished to afford every opportunity for the increase of foundlings, whom he regarded as a source of future armies, free from all the evils of conscription. A clause by which the emperor placed them at the disposal of the minister of the marine on attaining their twelfth year, thus reducing them to a state of military servitude has never been carried into effect. But, previous to the recent modifications in the law, the institutions for the support of foundlings were, in all other respects, regulated in accordance with the decree of 1811.

Various causes have concurred of late to excite enquiry into the working of these institutions in France. The attention of some has been arrested by the evidence of a growing deterioration of morals which is afforded by the progressive increase in the number of foundlings. The political economist has been alarmed by an annual expenditure of eleven millions

* Gaillard, p. 93.

† The Tours or turning boxes are an Italian invention of the time of Pope Sixtus IV. Their use was for many years confined to the hospice at Rome, which thence derived its name *Della Ruota*. In France they are formed by a hole in the wall of the hospice, closed with double doors, between which a cradle is placed. The act of opening the outer door to deposit a child in the cradle causes a bell to ring which gives notice to the attendant in the hospice. Or sometimes a cylindrical box placed perpendicularly and turning on a pivot is used, and this was the original form of the Tour.

of francs, which burdens the country, and leaves no money disposable for other purposes: and the physician, looking at a different feature in the case, sees equal cause for regret in the fact that, notwithstanding all this expense, the mortality is almost as great as it was fifty years ago, and that nearly 60 per cent. of the children die within a year after birth.

The works at the head of this article are some among many which have been written in order to explain these startling facts, to point out the sources of the growing evil, and to suggest a remedy. They were elicited by the offer of prizes made by various societies in France for the best essays on the subject. The treatise of MM. Terme and Monfalcon received in 1838 the honorable distinction of the Monthyon prize of the French Academy. The essay of M. Remacle and that of the Abbé Gaillard have each likewise been crowned by other societies. The work whose title stands first on our list seemed to us to claim that position by the diligence and research which it displays, by the thorough acquaintance of its authors with all the practical details of the subject, by their clear arrangement, lucid and elegant style, and by the candour with which they treat the opinions of others. The treatise of M. Remacle likewise possesses very considerable merit, though it must yield in completeness to the other two, since some points, such as the internal organization and hygiene of an hospital, the circumstances influencing the mortality of the children, &c., are altogether omitted, and others are treated of but very slightly. It is to the mode of admission into the hospital that he especially directs his attention, and, in common with M. Terme and his coadjutor, he decides most positively against the *tours* being any longer employed. The Abbé Gaillard appears as the warm apologist of the *tours*, the maintenance of which he looks upon as absolutely essential to the utility of foundling hospitals. His book displays great research, and contains much valuable information, though not always well arranged. He too supports his opinions by statistical tables, but these are often composed of numbers too small to be entitled to much weight, or are open to the charge of telling only part of the truth. But, in spite of these drawbacks, it is a most valuable storehouse of facts on the subject of foundlings, and reflects great honour on its author.

We are so used in this country to hear foundling hospitals stigmatized as absurd and mischievous in the extreme, that it may at first excite our surprise to find none of these gentlemen proposing to do away with them. An establishment "which may safely be termed a great public nuisance leading to unchaste life and to child-murder, beyond any other invention of the perverted wit of man,"* could hardly have been expected to find an apologist. But we shall see in the sequel that both of these charges are unfounded, and that many of the objections made to these institutions apply to their maladministration rather than to any vice necessarily inherent in them. We do not advocate the establishment of foundling hospitals; on the contrary, we believe them to be injurious, because they have no influence on those evils which they were instituted to prevent; because the relief they proffer can be obtained only by a deliberate sacrifice of the best feelings of our nature; because they are liable to abuses which it is almost impossible to prevent; and because, while they entail a

* Edinburgh Review, No. 76, p. 440.

great expense upon the country, they preserve the lives of but a very small proportion of their inmates.

Our limits will not allow us to investigate the subject in all its bearings. We will therefore endeavour to give our readers a sketch of its most important features, taking for our authorities, as we have done hitherto, the works whose titles we have given above, except when by references at the foot of the page, we intimate that we have derived our facts from other sources.

The point which will naturally first claim our attention is the extraordinary and progressive increase in the number of children at the charge of public charity in France. This number, which was estimated by M. Necker at 40,000 in the year 1784, amounted in 1798 to 51,000; had reached to 97,919 in 1818; and on January 1, 1834, had actually increased to 129,669. But this fact, at first so overwhelming, wears a much less grave aspect if we bear in mind, that before the year 1789 the number of children at the charge of the Lords Justices was unknown, no record of them being kept. The public, too, had then no legal claim to the benefits of the hospices, which were supported by the charity of private individuals or of the church, or by occasional grants from the king. Matters were of course greatly changed when the hospices were converted into national institutions, and when instead of being confined to a few large towns several were established in every department of the country. Moreover, it is the number of children admitted every year into the hospitals, not of those annually at the charge of the state, to which attention should be directed. The former alone illustrates the moral condition of the people; the increase of the latter may be simply indicative of the diminished mortality of the foundlings. Before the year 1824, however, no official documents stated the annual number of children exposed, as compared with the total births in the country, and we are now furnished with the results only of the decennial period from 1824 to 1833.

| Years. | | Legitimate Children born in the whole of France. | Births of Illegitimate Children. | Total number of Foundlings and Deserted Children admitted into the Hospices. |
|---|---|---|---|---|
| First quinquennial period. | In 1824 | 912,978 | 71,174 | 33,792 |
| | 1825 | 904,594 | 69,392 | 32,278 |
| | 1826 | 920,720 | 72,471 | 32,876 |
| | 1827 | 909,428 | 70,768 | 32,504 |
| | 1828 | 905,843 | 70,704 | 33,749 |
| | | 4,553,563 | 354,509 | 165,199 |
| Second quinquennial period. | In 1829 | 895,176 | 69,351 | 33,141 |
| | 1830 | 898,577 | 69,247 | 33,431 |
| | 1831 | 915,298 | 71,411 | 35,884 |
| | 1832 | 870,509 | 67,677 | 35,435 |
| | 1833 | 898,485 | 71,468 | 33,191 |
| | | 4,478,045 | 349,154 | 171,082 |
| Total of the 10 yrs. | | 9,031,908 | 703,663 | 336,281 |

The proportions resulting from this table are:

| FOR THE FIRST FIVE YEARS. | FOR THE LAST FIVE YEARS. |
|---|---|
| 1 illegitimate birth to 13·85 total births | 1 illegitimate birth to 13·83 total births. |
| 1 child abandoned to 29·71 do. | 1 child abandoned to 28·22 do. |
| 1 child abandoned to 2·15 illeg. births | 1 child abandoned to 2·04 illegitimate births. |

The increase then in the annual number of foundlings is not only very slight when compared with the population, but occurred almost entirely during the two years of trouble which followed the revolution of 1830: we shall hereafter see that times of distress or famine almost invariably produce a similar effect.

The table we have just given shows that during 10 years there has been no perceptible increase in the proportion of illegitimate children to the population throughout France, and the past 30 years yield nearly the same result. The works before us indeed all concur in stating that the proportion of illegitimate children is much greater now than it was at the beginning of this century. M. Remacle estimates their number at one in 20 of the total births at the beginning of this century instead of one in 13 or 14 as at present. Even allowing this to be the case, we do not think that any conclusion unfavorable to the morality of the French people could fairly be deduced from it. The conscriptions which drained France of its young men during the first ten or twelve years of the nineteenth century, would sufficiently account for the small proportion of illegitimate children when compared with the numbers presented in after years. But we do not hesitate to reject the numbers upon which this calculation is founded, for Sir Francis d'Ivernois* has shown how little they are to be trusted. It appears from that gentleman's researches, that the official documents respecting the population under the consular and imperial governments are not entitled to any credence. They were deduced from registers of two millions of individuals only, to which a process was applied founded on some erroneous notions of Laplace. These results, though proved to be incorrect by a report made to government in 1801, and afterwards published without authority, as well as by the census of the population, were nevertheless printed as official documents. The reason of this was that they favoured an extraordinary and successful *persiflage* of Napoleon, who persuaded the French that wars and conscriptions had a direct tendency to multiply marriages and to increase the population of the country. Since the year 1816 the number of illegitimate births has continued nearly stationary at 1 in 14 or 7 per cent., while the increased number of marriages, with the smaller proportion of children to each, and the diminished mortality, point to an improved moral as well as physical condition of the population.†

But it will become still more evident how little connexion exists between illegitimacy and the desertion of children, if we compare the number of illegitimate children in different departments of France.

* Sur la Mortalité proportionnelle des Populations Normandes considérée comme mesure de leur aisance et de leur civilisation, (Tiré de la Bibl. Univ.) Genève, 1838. See especially the Notice Supplémentaire.

† See the researches of Sir F. d'Ivernois, Sur la Fécondité et la Mortalité proportionelles des Peuples considerées comme mesures de leur aisance et de leur civilisation. (Tiré de la Bibl. Univ. Août, 1835.) Genève, 1836. In this paper he points out the true interpretation of the above-mentioned facts, and explains what at first sounds so paradoxical.

| Departments. | Date. | No. of Illeg. Births. | No. of Foundlings. |
|---|---|---|---|
| Bouches du Rhone . | 1832 | 1219 | 825 |
| Charente Inférieur . | 1824 | 582 | 573 |
| Corsica . . . . . . | 1824 | 141 | 143 |
| Cher . . . . . . . | 1827 | 391 | 411 |
| „ . . . . . . . | 1828 | 354 | 377 |
| Dordogne . . . . . | 1829 | 578 | 755 |
| Ille et Villaine . . . | 1832 | 312 | 466 |
| The following table gives an opposite result: | | | |
| Jura . . . . . . . | 1826 | 588 | 68 |
| „ . . . . . . . | 1828 | 452 | 58 |
| Moselle . . . . . . | 1824 | 720 | 78 |
| Haut Rhin . . . . | 1826 | 1221 | 34 |
| „ . . . . . . | 1832 | 980 | 78 |
| Seine et Oise . . . | 1826 | 822 | 30 |
| Haute Saone . . . . | 1824 | 1060 | 8 |
| „ . . . | 1829 | 1434 | 9 |
| „ . . . | 1831 | 841 | 3 |

The preceding tables are formed from the statements of MM. Terme and Monfalcon, p. 353; but the fact has not escaped the attention either of M. Remacle or of the Abbé Gaillard. The Baron de Gerando* likewise, in his valuable work on public charity, illustrates the same subject. It results from his investigations that in 4 only of those 12 departments in which foundlings are most numerous, do the illegitimate births exceed the average number. The converse also holds good, and 2 only of 12 in which the number of foundlings is smallest, present less than the average number of illegitimate births. The same fact will be seen to be still further elucidated by a comparison of the proportion of illegitimate births per 1000, in countries with and in those without hospitals for foundlings:

| STATES WITHOUT HOSPICES. | Illegitimate Births. | STATES WITH HOSPICES. | Illegitimate Births. |
|---|---|---|---|
| Prussia . . . . . | 69 per 1000 | France . . . . . | 71 per 1000 |
| England and Wales . . | 55 „ | Naples . . . . . | 46 „ |
| Wales alone . . . | 83 „ | Archduchy of Austria . | 42 „ |
| Saxony . . . . | 121 „ | | |
| Hesse . . . . | 149 „ | | |

An investigation into the causes which render the proportion of illegitimate births so different in the several departments of France, or in those countries which figure in the above table would take us far beyond our limits. We do not infer from these facts that chastity is less valued in Protestant than in Catholic countries, but we seek simply to show that the want of chastity among women is not the cause of children being deserted, and that it is not to the existence of foundling hospitals that the increase of illegitimate births in any country is to be attributed. Public opinion regards the circumstance of an unmarried female becoming pregnant very differently in different countries. In some countries, as in the isle of Portland or in the neighbourhood of some of our collieries, a girl's pregnancy is merely a preliminary step to her marriage; while in other places such an occurrence would ruin her character and blight her prospects. Some persons too live in a state of

* De la Bienfaisance Publique, Paris, 1839, 8vo. Tome ii. p. 249.

concubinage simply because, owing to their distance from home, they are unable to procure those documents which, in France as well as in many German states, are indispensable to their marriage. This is a cause which tends to swell the number of illegitimate children in many of the large manufacturing towns of France, and has led to the establishment of charitable institutions, such as the society of Saint Regis at Paris, for facilitating the marriage of the poor.

It is our object, however, to enquire not into the causes of bastardy, but into those circumstances on which the exposure and desertion of children depend. It is commonly said that the immorality of a people is the cause of illegitimate births, but that misery leads them to desert their children. At first sight no truth could appear more self-evident than the latter clause of the above statement, for it is a well-known fact that the number of foundlings increases in seasons of famine or at times of unusual distress. In the year 1817, for instance,* the number of foundlings admitted into the hospices of Belgium rose from 3000 to 3945. In Paris the admissions of foundlings in that disastrous year increased by 800, and by 5000 throughout the whole of France. The same has been observed to take place during winters of unusual severity. The number of children admitted into the hospice at Paris during the three winter months of 1803, amounted to half the total admissions in that year, and the greatest number of children were exposed in the last of those three months, when the working classes had altogether exhausted their resources. But, though seasons of unusual distress may be attended with a sudden increase in the number of foundlings,† it does not necessarily follow that we can generalize that fact, and refer the desertion and exposure of children to the pressure of poverty on a people. M. Remacle, following M. Villeneuve, has given at p. 167 some very elaborate tables, displaying the proportion of foundlings to the population in comparison with the number of persons receiving public relief. On dividing France into three districts or zones, the suffering, the intermediate, and the prosperous, we obtain the following results:

| | Suffering District. | Intermediate District. | Prosperous District. |
|---|---|---|---|
| Proportion of indigent to the population | 1 to 15 | 1 to 23 | 1 to 37 |
| „ foundlings „ | 1 to 345 | 1 to 488 | 1 to 601 |

M. de Gerando,‡ however, remarks with great truth that the number of recipients of public charity is by no means an accurate index of the amount of want in any particular district. If, for instance, we divide the whole of France into seven districts, it will be seen that the districts in which are the greatest number of paupers is that likewise in which the proportion per 1000 paying taxes is highest, and that the number of foundlings does not bear any fixed relation to either of these supposed signs of the well-being of a country.

* Quetelet et Smits, Recherches sur la Reproduction et la Mortalité de l'Homme. Bruxelles, 1832. 8vo, pp. 66.

† MM. Terme and Monfalcon show, p. 191, by the results afforded by the foundling hospital at Lyons in 1836, a year of extraordinary distress in that city, that times of unusual want are not invariably attended with an increase in the number of desertions of new-born infants; poverty has much more to do with the abandoning of children a few years old than with the desertion of new-born infants.

‡ Lib. cit. tom. ii. p. 253.

| Districts. | Number of Indigent per 1000. | Proportion of Inhabitants per 1000 paying Taxes. | Number of Foundlings per 1000 Births. | Number of Foundlings per 1000 Natural Births. |
|---|---|---|---|---|
| North of France . | 69 | 42 | 43 | 408 |
| Ditto, exclusive of the department of the Seine . . . | 70 | 42 | 25 | 355 |
| East . . . . | 37 | 38 | 24 | 326 |
| Centre . . . . | 38 | 29 | 34 | 584 |
| South . . . . | 42 | 32 | 35 | 628 |
| West . . . . | 48 | 26 | 29 | 600 |
| Corsica . . . | 21 | 18 | 28 | 645 |
| The whole of France . | 49 | 34 | 33 | 469 |

Besides, if poverty were the cause of the progressive increase in the number of foundlings, we must necessarily admit that the condition of the lower orders in France has continued to deteriorate during the past twenty-five years, while the contrary is, we believe, universally allowed to be true.

If then the increased number of foundlings cannot fairly be attributed either to a growing corruption of morals, or to the pressure of poverty, there remains no other explanation than that suggested by M. Quetelet's table which we have already quoted, namely, that it depends on the increase of the population. This fact, though so obvious, seems not to have been apprehended by any one before it was pointed out by MM. Terme and Monfalcon. They bring forward evidence for which we have not space, to prove that "the general law of increase in the desertions of new-born infants is the augmentation of the population. When the population becomes more considerable, the hospices receive a larger number of foundlings; when it diminishes the number of desertions immediately undergoes a perceptible reduction." (Terme and Monfalcon, p. 202.)

But the fact that the number of new-born infants deserted annually continues undiminished is of importance sufficient to claim the attention of a wise and enlightened government. All efforts to check the evil, however, must be fruitless so long as the system of secret admission into the hospices prevails. We shall have occasion to see many proofs of its injurious tendencies, but one of its worst results is shown in the large proportion of children born in wedlock who are sent to the hospice by their parents. Their number has been estimated by M. Remacle (p. 190) at 1 in 10 in Paris and most departments, and even as high as 1 in 5 in some cities. M. Gaillard observes (p. 143) that the proportion of 1 in 10 is very low, being only 1 in 270 legitimate children; a number the desertion of which may he thinks be fully accounted for by accidental causes, such as the destitution of the parents. He seems, however, to lose sight of the fact that a large proportion of these children are deposited in the *tour* without any certificate of their birth, and MM. Terme and Monfalcon (Nouvelles Considérations, p. 56,) estimate at 2400 the number of legitimate children thus annually bastardised by their unnatural parents.

In the chamber of Deputies, and at the meetings of the Société de la Morale chrétienne, M. de Lamartine has declaimed eloquently on the moral impossibility of a married woman deserting her child, and M. Gaillard assures us that it is an act of which none are guilty, but when impelled to it by extreme distress. MM. Terme and Monfalcon tell a different tale:

"In large manufacturing districts and among the artisans, the fathers and mothers separate themselves from their children with a most lamentable carelessness, and look on it as infinitely more convenient and desirable to take their children to a hospice and to forget them than to trouble themselves about bringing them up. M. Lelong, a member of the general counsel of Seine Inférieure, states that in some neighbourhoods the number of foundlings has equalled, and sometimes even exceeded the number of children born out of wedlock." (p. 197.)

Their own experience supplies them with the following striking fact, which will be found detailed at length in the Nouvelles Considérations, (p. xxviii.): At the Hôtel Dieu of Lyons there is a lying-in ward for the wives of the artisans, in which from 500 to 600 are delivered annually. More than 60 of these women are detected every year by the vigilance of the police in the attempt to send their children to the hospice, and are compelled to take them back again. Many, however, elude the officers and succeed in getting rid of their children, so that it is not too high an estimate to calculate at 100 out of 500 the number of legitimate children bastardised or sought to be bastardised by their parents, while it is very seldom that the women who thus abandon their children are the poorest. Two thousand children are admitted every year into the hospice at Lyons: 800 of these are the children of women delivered in the hospice; 200 are children above three days old, placed in the *tour*, and presumed to be legitimate; 200 more are from one to ten years old, and are likewise supposed to be legitimate, and the remaining 800 are new-born infants deposited in the Tour, and consequently regarded as illegitimate. Of 1389 children restored to their parents at Lyons during eight years, either by direction of the municipal authorities, or at the parents' request, 424 or one third were legitimate. Many more facts of a similar kind might be adduced if we had space; but enough, we think, has already been said to prove that the number of foundlings in France is swelled by many being at the charge of the state who in reality have no claim upon the public beneficence.

The French government is now fully alive to the evils which result from the want of all control over the admission of infants into the hospices; and accordingly the reduction of the number of *tours* forms a main feature in the measures which have been recently introduced with the view of checking the desertion of children. It will appear in the sequel that very beneficial results have followed even from a very partial adoption of these plans; but the arguments of those who oppose any alteration being made in the mode of admission into the hospice are of such a nature as to merit a careful investigation.

When the propositions of the government were first brought forward in the chamber of Deputies, they were met by such violent opposition, that a stranger might have imagined the employment of the Tours in

France, to have been at least coeval with St. Vincent de Paul, instead of which they were not generally adopted until 1811. By some strange misconception too, the system of secret admission has come to be regarded by many as an essential feature of foundling hospitals, and as such it has been attacked by some and defended by others. Others, indeed, to whom belong MM. Terme and Monfalcon, M. Remacle, and the Baron de Gerando, distinguish between opposition to the *Tour* and to the *Hospice*; they praise the latter, but use every argument against the former.

The advocates of the *tours* applaud them most for that profound mystery in which they involve every circumstance connected with the desertion of children. "Ingenious invention of Christian charity," says their most eloquent apologist, M. de Lamartine, "which has hands to receive, but neither eyes to see, nor tongue to tell." It screens the mother, say they, from harsh and unjust reproaches, and removes the grand inducement to infanticide by providing a place where her child and her shame may both be hidden. If a child were never abandoned except when misery or an overwhelming sense of shame impelled the mother to desert it, then indeed there might be an advantage in this impenetrable secrecy; but the facts we have already quoted from MM. Terme and Monfalcon prove the case to be far otherwise. M. de Gerando* furnishes us with data from which we may approach to a right estimate of the moral condition of the persons who people foundling hospitals. During the twenty years from 1816 to 1835, 57,400 women have been delivered in the Maternité at Paris, and $\frac{1}{2}\frac{9}{0}$ of them sent their children to the Hospice des Enfans Trouvés. Of these women it is calculated that about a quarter were seduced by promise of marriage, and that they might be reclaimed to a virtuous course of life; another quarter had already begun a vicious life, but possibly might be reclaimed; and the remaining half consisted of women more or less depraved, but with regard to whom all attempts at reformation may be regarded as useless.

It has been asserted that the *tours*, by removing the temptation to infanticide, prevent the commission of that crime; but this statement is utterly erroneous. We have already shown that uo connexion exists between the proportion of illegitimate births and the desertion of children; and it is not less demonstrable that infanticide and the desertion of children depend on causes altogether different. If the statements of those who advocate the system of secret admissions were correct, the number of *tours* in a department and the number of infanticides would be in directly opposite proportions to each other; while infanticides should prevail most in countries where foundling hospitals are unknown. Now M. Remacle gives a table (p. 215), of which the following are the results:

| No. of Departments. | No. of Inhabitants. | No. of Foundlings. | Proportion of Foundlings to Inhabitants. | No. of Tours. | No. of Infanticides in 4 years. |
|---|---|---|---|---|---|
| 10 | 3,114,978 | 19,702 | 1 to 157 | 30 | 26 |
| 10 | 3,916,093 | 3307 | 1 to 1183 | 34 | 29 |

* Op. cit. tome ii. pp. 257-61. The statements there given with reference to the occupations, &c. of these persons well deserve attention.

If we compare different countries with each other, as the following table, (Remacle, p. 226,) enables us to do we shall find a greater proportion of infanticides in those countries where *tours* exist.

| COUNTRIES WHICH HAVE TOURS. | | Inhabitants. | COUNTRIES WITHOUT TOURS. | | Inhabitants. |
|---|---|---|---|---|---|
| France . . . | 1 in | 326,530 | England . . | 1 in | 855,903 |
| Belgium (Brabant, etc.) | 1 in | 439,768 | Belgium (Liege, etc.) | 1 in | 546,648 |
| Ireland . . | 1 in | 287,566 | Grand Duchy of Baden | 1 in | 228,020 |
| | | | Prussia* . . | 1 in | 76,873 |
| Average . . | 1 in | 351,321 | Average . . | 1 in | 426,861 |
| | | | Average (exclusive of Prussia) . . | 1 in | 543,524 |

A still stronger proof of the truth of this assertion is afforded by the fact that the suppression of the *tour* in many provinces of Belgium has not been followed by any increase in the commission of infanticide. So far indeed was this from being the case that the closure of the *tour* in Maestricht was immediately followed by a diminution in the number of infanticides within the district. Of this result, which at first sight may appear so extraordinary, the following is, we doubt not, the correct explanation:

"I had long," says M. Schaetzen, in his official report to the Belgian Council of State on March 10, 1834, "entertained the idea that foundling hospitals had a necessary tendency to preserve the lives of new-born infants. I endeavoured therefore to explain this occurrence (the diminution of infanticides after the Tour at Maestricht had been closed), and to ascertain the reasons why infanticides had not increased in the province of Limburg in inverse proportion to the number of infants deposited in the foundling hospital, and my researches have led me to a solution of the problem. I discovered that the crime of infanticide was not committed on children who had lived a few days; that as soon as any woman had experienced the pleasures of being a mother, she no longer thought of attempting the life of her child; that this barbarous act was committed only during the first moments of the woman's embarrassment, and while she was still struggling between the feeling of shame and of natural affection; and lastly, that the life of the child was safe whenever the mother was sure that the fact of her having been delivered was known to a second or third person."† (Remacle, p 233.)

M. Schaetzen's reasoning holds good with reference to all those cases of infanticide in the commission of which the sense of shame has any part. The crime, however, is perpetrated less frequently by young girls overwhelmed by the dread that their frailty will be exposed, than by women well schooled in vice, and who have long been dead to all feeling of shame. MM. Terme and Monfalcon assure us, "That those who are guilty of this crime are not young girls; but that two thirds of 336 per-

* There is reason to believe that some error exists with reference to the proportion of infanticides in Prussia; and M. Remacle expresses great doubts as to its correctness.

† The diminution of infanticides in Limburg must have been in a great measure owing to the temptation to a woman to conceal her pregnancy having been removed by the suppression of the *tour* at Maestricht.

sons accused of infanticide were women between twenty-five and thirty-five years of age; most of whom had not received any education, while many before committing infanticide had sunk to the lowest depths of depravity and infamy."—(Nouvelles Considérations, p. lxxvii.)

In some departments of France the experiment of suppressing the *tours* has been tried; and, as the friends of the measure say, with good success. This statement, however, has been contradicted by others, who point to statistical tables to show that since attempts have been made to do away with the plan of secret admissions, infanticide has grown much more frequent throughout France. From these documents* it appears, that during the three years previous to the introduction of the new measures, eighty-two cases of infanticide were annually sent for trial at the assizes, but that the average has been as high as 117 during the three years 1834, 1835, and 1836, subsequent to the change. This fact would seem to lead inevitably to the conclusion that any diminution in the number of foundlings has been purchased at the expense of the lives of very many infants.† There are however many sources of fallacy in the grounds on which this conclusion is founded. Had M. Smith, instead of confining his observations to the years of disturbance which followed the revolution, when the data afforded us are likely to be more or less incorrect, extended his researches some years back, as far for instance as 1826, he would have obtained an annual average of ninety-five infanticides instead of eighty-two. Much of this apparent increase too must be attributed to the greater activity of the police, and the more able administration of justice. During the four years preceding the suppression of the *tours*, the proportion of prisoners charged with infanticide who were sent to take their trial at the assizes, was to those discharged for want of evidence as 37 : 100; while during the four following years the number was as 43 : 100. The acquittal of those sent to trial have likewise fallen from 50 per cent. to 42 per cent. Moreover, in two-thirds of the departments of France, the frequency of infanticide is in direct proportion to the frequency of other crimes. The following table too, drawn up from materials contained in a paper by M. Bayard,‡ will show clearly that we must look to some cause far other than the feeling of shame, to account for whatever increase in the frequency of infanticide has actually taken place.

* Rapport sur les Enfans Trouvés, par M. Smith, 8vo, p. 30.

† In the Fifth Report of the British Association is a paper by Professor Maunsell on the Statistics of the Dublin Foundling Hospital. It is drawn up in such a way as to serve as an apology for the institution, since the suppression of which, Dr. Maunsell alleges that infanticide has greatly increased in frequency. Now, to say nothing of the exceedingly imperfect returns on which this statement is based, it should be observed that they do not go further back than the year 1829; while the system of secret admission was done away with, and the number of admissions was limited to 500 annually in 1822. Since that year, therefore, the supposed influence of the institution in checking infanticide must have ceased, and Dr. Maunsell's facts must consequently be regarded as altogether irrelevant to the question which he was desirous of elucidating.

‡ Considérations médico-légales sur plusieurs cas d'infanticide, et sur le fréquence de ce crime. Annales d'Hygiène, tom. xxiv. p. 331. It contains much valuable statistical information.

| | | During four years (1830-33), immediately before the Suppression of any *Tours.* | During four years (1834-37), immediately following the Suppression of some *Tours.* |
|---|---|---|---|
| Proportion of persons involved in 100 cases of infanticide . . . | | 109·3 | 112·4 |
| Proportion of males to 100 females . . . | | 5·3 | 7·4 |
| Proportion per cent. of the criminals . | under . 25 | 34·9 | 35·9 |
| | from 25 to 35 | 42·1 | 38·7 |
| | from 35 to 45 | 17·3 | 16·6 |
| | above . 45 | 5·6 | 8·8 |

From this table it appears that the cases in which two persons are concerned in the commission of the crime have increased in frequency; that males are implicated in it oftener than formerly; and that the great increase has taken place among persons considerably advanced in life. These are circumstances which indicate a great deterioration of morals, but which likewise show that the habitually vicious are those who have principally contributed to make the crime of child-murder more frequent. As a further illustration of this fact, may be mentioned, (Remacle, p. 231,) that of 810 persons brought to trial for infanticide in the course of eight years, 169 were married, and 146 had children then living, and that nine men and eighty-two women were either living in concubinage or were the parents of bastard children at the time they committed the act. It is likewise worthy of remark, that 697 of these 810 could neither read nor write.

The preceding facts will, we think, make it appear very doubtful how far any increase of infanticide can be attributed to the suppression of the *tours*; but there exists still stronger evidence that such has not been the case.

"Twenty-four departments have suppressed forty-eight *tours* in less than two years, and in only nine of these has any increase in the number of infanticides occurred in the year 1835, while in thirteen they have become less frequent, and in the remaining two their number has continued unchanged. Thirteen departments have suppressed more than one *tour;* in five of these the number of infanticides has increased, and in seven has diminished. Eight departments did away entirely with their Tours, before January 1, 1834. Five of these have had fewer charges of infanticide than usual in the two following years; one only has presented any sensible increase; for the other two the results of the two years tally exactly with those of previous years.

Total increase . . . 5·8
Total decrease . . . 6·7

"The above are the results afforded by those departments which have suppressed their *tours*: fifty-four have preserved their number unchanged. Twenty-five of these have had fewer infanticides in 1835, in twenty-nine they have become more numerous. The total diminutions have amounted to 18·5; the total increase has been 40·3." (Remacle, p. 218.)

But the *tours* not only do not diminish the number of infanticides, but there is reason to believe that they are an indirect cause of the death of children; partly by affording a temptation to unmarried women to conceal their pregnancy, partly by inducing persons to desert their children who would never think of so doing unless they were certain of their wickedness being concealed by a veil of impenetrable mystery. Nor is this all the evil which they occasion, but infants are brought from other countries to all the hospices near the frontier; and MM. Terme and Monfalcon, (Nouvelles Considérations, p. lxxii,) inform us, that a large proportion of the foundlings received into the hospice at Lyons are sent from Valais, from the canton of Fribourg, from Geneva, and from Savoy. There are persons who earn a livelihood by carrying children across the frontier and depositing them in the *tour* of some French hospice. For this, twenty francs used to be their charge, but competition has now reduced it to fifteen; and it is a well-ascertained fact that these messengers frequently lighten their labour by destroying the children whom they have undertaken to convey to a hospice.

The last argument against the suppression of the *tours* which we shall notice is, that where the measure has been adopted it has not led to any diminution in the number of foundlings. This assertion is based on a very small number of facts, to which quite as many of an opposite character might be opposed. The results, which have been recently obtained in Paris, prove, as will presently be seen, how much may be done by a wise administration to diminish the frequency of the desertion of children, and the following table of the number of foundlings in Geneva at different times, shows to how great an extent the evil may be either fostered or checked by the different plans pursued by the legislature.*

| | Foundlings. | |
|---|---|---|
| | Annual mean. | Proportion per 1000 births. |
| During the whole of the French regime from 1799 to 1813 | 37 | |
| From 1806 to 1812, a time when the foundling hospital on the French system was in full activity . . . | 45 | 78 |
| Since the restoration, from 1814 to 1823 . . . | 9 | 17 |
| ,, from 1824 to 1833 . . . | $2\frac{8}{10}$ | 4 |

It has never been proposed to modify the present system suddenly, nor to do away with the Tours throughout France, without providing a substitute for them.† This substitute MM. Terme and Monfalcon and M. de Gerando think they have found in the system of open admission, *à bureau ouvert*. It is by no means a novel plan, but one which has been adopted in Paris for the last twenty years, and has worked so well that no difficulty is ever experienced in obtaining the address of any woman whose child is brought for admission, while though the *tour* has continued open, the number of children deposited in it scarcely exceeds one a month. It is proposed to multiply the number of depôts where foundlings may be received, and to render them much more numerous than the *tours* ever were. At any of these depôts a child will be received on

* Bibliothèque Universelle de Genève, t. x. p. 59.

† We purposely omit giving an account of the experiment of *Deplacement,* since it was not persevered in. It consisted in the removal of foundlings from their nurses in one department to others in a different department, or in a sort of periodical exchange of the children. It was adopted in order to check an abuse which had become very frequent, that of women sending their children to the *tour* of a hospice, and afterwards contriving to obtain charge of them again, as nurses paid by the institution. The above means at once produced a great reduction in the number of foundlings; but many circumstances concurred in rendering it so unpopular, that it was not thought advisable to continue it.

the name, address, &c. of its mother being stated, but none will be received unless this information is afforded; and a refusal to give it will be regarded as a proof of the unfitness of the applicant to partake of the benefits of the institution. No one but the mayor of the place will receive the application for admission; there will therefore be no compromise of any one's character; and if secrecy is strictly observed with reference to all persons who are admitted into the Maternité at Paris, there is no reason to fear that in this instance it will be violated. A stop will by these means be put to the importation of children from other countries, and that great abuse, the secret admission of legitimate children, by which it is computed that 2400 are annually bastardised, will be done away with.

It was likewise proposed to relieve poor women at their own homes, and thus to enable those to bring up their children who might otherwise be driven by poverty to abandon them.

Highly satisfactory results have been obtained at Paris by granting relief to mothers in order to prevent them from sending their children to the hospice, and by exacting a promise from all women received into the Maternité to suckle their infants while there, and to take them out on their departure from the institution. In the course of the first eight months after the adoption of these measures, the mothers of 458 children have been induced, by kind persuasions and by some pecuniary assistance, to retain their infants whom they had intended to abandon. One of the most gratifying features too in the working of this system is that the deaths among the infants whom their mothers were persuaded to keep charge of, amounted only to 1 in 14, while the mortality among the foundlings was 1 in 3. The following statement is given by the Baron de Gerando* of the working of these measures after they had been fourteen months in operation:

"The year 1838, when compared with the seven preceding years, has yielded the following results:

| | Mean of the Seven former years. | The year 1838. |
|---|---|---|
| Children admitted . . . . | 4929 | 3037 |
| Died in the hospice . . . . | 1306 | 763 |
| Sent to the country . . . . | 3651 | 2277 |
| Claimed by their relations . . . | 39 | 28 |

"During the course of the same year, 1838, although the *tour* has remained constantly open, the number of children deposited in it has not exceeded 60. The number of children exposed in the public thoroughfares during the course of the year was 28; and the number of children deserted in the entries of houses or in their interior was 11. The data collected in the Maternité during the seven years prior to 1838, compared with those of 1838, have given the following results:

| | | Mean of the Seven former years. | The year 1838. |
|---|---|---|---|
| Women | admitted . . . | 2960 | 3153 |
| | delivered . . . | 2642 | 2946 |
| Children of Women delivered in the Maternité . | still-born . . . | 144 | 144 |
| | died in the hospice . | 72 | 119 |
| | retained by their mothers . . | 717 | 1437 |
| | abandoned by their mothers in the hospital | 1751 | 1297 |

* Op. cit. t. ii. p. 582, bis.

"Thus two fifths of those children who would probably have been abandoned to public charity, if the government had not taken steps for applying those regulations which are sanctioned by law, have by these measures been preserved to their mothers and families."

And this improvement, be it observed, has been accomplished even without closing the *tour*. It has not increased the number of infanticides, nor given rise to the desertion of more children in the public thoroughfares than in previous years, and it has been attended with a considerable saving in expense. For details concerning these points, however, we must once more refer to the valuable work of Baron Gerando.

It still remains for us to examine into one of the most important questions connected with foundling hospitals, namely, the high rate of mortality among their inmates. The mortality of early infancy varies greatly in different countries, being as low as 14·6 per 100 births in England and Wales for children under one year, and 21 per cent. for children under five years.* As a contrast to this we may mention some of the Eparchies of Russia, as Nischni Nowgorod, where 31 per cent. die during the first year: and according to Herrmann's researches the mortality during the first twelve months of existence averages 27 per cent. throughout the whole Russian empire.† We shall probably not underrate the mortality throughout Europe during the first year by estimating it at 21 per cent.; the average yielded by data furnished by Burdach‡ which refer to five different countries. But if with this we compare the mortality in foundling hospitals, we shall be forced to allow that they have signally failed in one great object of their institution, the preservation of the lives of infants.

The great mortality among foundlings is not observed merely in one country, nor is it owing to defective arrangements peculiar to some hospices, but is alike common to all. The proportion of deaths at Turin is estimated at 75 per cent. of the total admissions; at Moscow the admissions during ten years averaged 5255 annually, and the deaths 3471. During the ten years from 1822 to 1831, 39,114 children were received into the hospital at St. Petersburg, and 31,779 died,§ and MM. Terme and Monfalcon state that 377 died out of 417 who were admitted into the hospital at Archangel. At the close of the last century the mortality of foundlings during the first year,|| was

| | |
|---|---|
| At St. Petersburg . . | 40 per cent. |
| Florence . . . . . | 40 |
| Barcelona . . . . . | 60 |
| Paris . . . . . . | 80 |
| Marseilles . . . | 90 |
| Dublin . . . . | 91 |

In the year 1824, M. Chateauneuf¶ stated the mortality of foundlings

* Second Report of the Registrar-General, p. 10.

† Lichtenstädt, Ueber die Ursachen der grossen Sterblichkeit der Kinder. Petersburg, 1837, pp. 18-30.

‡ Physiologie. Band iii. Erste Tabelle.

§ Notice sur quelques uns des établissemens de Bienfaisance du nord de l'Allemagne, et de St. Petersburg, par M. Leuret, in Annales d'Hygiène, t. xx. p. 346.

|| Considérations sur les Enfans Trouvés, par M. B. de Chateauneuf, Paris, 1824, 8vo, pp. 64.

¶ Lib. cit. Tableau ii.

throughout France during the first year, to be 57·6 per cent., and the statistical tables appended to the work of M. Remacle, show that after fourteen years it has not undergone any important diminution. We think, however, that M. Remacle is mistaken in supposing that during the past fifty years there has been an increase in this mortality. He asserts, on the authority of Raulin, that, in the year 1768, 75 per cent. of the foundlings at Grenoble were alive at the end of their seventh year; forgetting not only how little reliance can be placed upon statistical details before there was a regularly organized administration of foundling hospitals, but also that so low a rate of mortality as that just mentioned was never presented by the population of any country. It should too be borne in mind, when comparing the mortality of foundlings with that of the population generally, that in our estimate of the latter, we include the rich as well as the poor, and that a table of the mortality of the children of the poor during the first twelve months of their existence would bear a far higher proportion to the births than 21 per cent. It appears from a valuable paper by M. Villermé, "On the Mortality in the different quarters of Paris,"* that while the deaths of children under one year old form only 17 per cent. of the total deaths in the first arrondissement, which includes the most fashionable part of the city, the mortality among children of the same age in the twelfth, which is the poorest district, is so high as to form 25 per cent. of the total deaths, and 32 per cent. in the Rue Mouffetard, where the inhabitants are extremely poor. The influence of poverty too is still more strikingly exemplified by the fact, that in the Rue St. Honoré and Rue du Roule, which though situated in the twelfth arrondissement are not inhabited by poor persons, the mortality under one year is only 14 per cent. of the total deaths; and that the mortality from birth to the age of twelvemonths in the Rue Mouffetard is equal to that from birth to ten years in the Rue St. Honoré and Rue du Roule.

But, even though we were to compare the mortality of foundlings with that of children in the poorest parts of Paris, we should still find but little cause for satisfaction. MM. Terme and Monfalcon furnish us, p. 229, with tables of the mortality at different ages, of foundlings belonging to the hospice at Paris. It results from them that the mortality of foundlings, from birth to one year, is to the mortality in the nine subsequent years as 7·6: 13·7; or, in other words, that the mortality, from birth to one year, amounts to 66·8 per cent. of the total mortality from birth to ten years. It is well known to all persons who are at all familiar with statistical enquiries, that the rate of mortality during the first twelve months of existence, affords a very correct index to the general mortality of any people. We shall therefore confine our investigations into the causes of the mortality of foundlings to those which are in operation before they have completed their first year.

The first cause of mortality which we will mention is the transport of children from a distance, and their consequent exposure to the inclemency of the weather.

It is of course impossible to do more than guess at the distance which those children have been brought who are exposed in the *tours* of the French hospitals. Some means, however, appear to have existed by which

* Annales d'Hygiène, t. iii. p. 331.

it was ascertained in most instances how far children were brought to the Dublin Foundling Hospital. Mr. Wakefield gives a table in his work* on Ireland, from which it appears that 59 per cent. of all infants who had been brought a distance of more than 50 miles, died in the hospice, while the mortality among the other children did not exceed 48 per cent. The effects of this transport are much less sensible when the weather is warm than when the temperature is low. M. Gaillard has drawn up a table (p. 162,) showing the mortality of foundlings deposited in the *tour* of the hospice at Poitiers, as compared with that of infants who were merely removed thither from the Maternité. It appears that during the six warm months of the year, 7 per cent. of the former die to 6 per cent. of the latter; but that during the six winter months the mortality of the former rises to 19 per cent., of the latter only to 10 per cent. It is probable that but few of the foundlings deposited in the *tour* at Poitiers are the children of inhabitants of the town, but that most are brought from a distance; for in Lyons, the *tour* of which must be in a great degree supplied from the city itself, the mortality of children placed in it is only 1 in 23, while 1 foundling dies of every 8 born in the Maternité. Two causes concur to diminish the mortality of children deposited in the *tour*. First, that while children brought from the lying-in hospitals are always new born, those deposited in the *tour* are frequently two or three days old, or even older. Second, that the women who are delivered in the lying-in hospitals are usually poorer, and in all respects worse off than those who after being delivered by midwives send their infants to the *tour*, and that consequently their children are more liable to disease.

The second and most important source of the mortality of foundlings is to be found in the various substitutes for the mother's milk, with which it has been attempted to feed them. But, important as it is to provide for infants the food which nature has destined for them, almost all foundling hospitals are essentially deficient in this respect. The fearfully high mortality in the Dublin Foundling Hospital is in a great measure attributable to the want of proper arrangements for providing wet-nurses for the children. Mr. Wakefield† says that at the time when he wrote his work, one woman very often had to suckle two infants, sometimes even five. The result of this system is shown by a mortality of 37 per cent. of the admissions within a fortnight after the children entered the hospice, and the mortality within this period formed 72 per cent. of all the deaths which took place during the stay of the infants in the hospice.‡ MM. Terme

* An Account of Ireland, Statistical and Political, by Edward Wakefield. London, 1812, vol. ii. 4to, p. 429.

† Op. cit. vol. ii. chap. xxiv.

‡ We cannot omit noticing a statement of Professor Maunsell in his paper on the Dublin Foundling Hospital, to which we have already alluded. He says that of 51,523 children admitted between the years 1798 and 1831, 12,153 died immediately on admission, and therefore ought not to be reckoned as forming a part of the mortality of the institution. We will not follow him in his subsequent reasoning, by which he arrives at the startling conclusion that the proportion of foundlings who attain their ninth year is greater than that of other children. Immediately, however, is a very vague term, and may be applied either to days or hours. If to the latter this statement is directly contradicted by the observations of Mr. Wakefield, who even adduces tables to "prove the inaccuracy of Mr. Tooke's opinion, that the loss of lives in foundling hospitals is to be ascribed to the weak state of the infant previously to admission." See Wakefield's Ireland, vol. ii. p. 427.

and Monfalcon mention, (Nouvelles Considérations, p. lxx.) that in the hospital at Madrid, where the mortality is likewise extremely high, one woman frequently suckles three infants.

The great difficulties in the way of obtaining wet-nurses for the foundlings have formed the chief inducement to the frequent attempts which have been made to bring them up by hand. M. Gaillard shall relate the result of one experiment:

"At Parthenay, in the department of Deux-Sèvres, of 153 foundlings, 54 died between the ages of one day and twelve months, or 35 per cent., which is a higher proportion than that presented by Poitiers. At X———, of 244 new-born infants, 197, or 80 per cent. had died by the end of the first year. Struck by the enormous difference which existed between this number and those of Poitiers and Parthenay I determined to investigate its cause. I ascertained that, in this hospice, as much attention is paid to the children, and the nurses are under oversight as strict as at Poitiers and Parthenay. But at X———, none of the infants are suckled, but all are fed; and the reason assigned for so doing is, the fear of infecting the nurses with syphilis. Be this as it may, I have been assured by many persons connected with the management of the institution, that the fearful mortality just mentioned can be attributed to no other cause than the practice of not suckling the children. The officers of the institution have tried all means to remedy this evil, but neither their own efforts nor those of some most excellent female assistants have been of the slightest service; and the only measure by which they could reduce the mortality was, the having recourse to suckling the children by wet-nurses." (Gaillard, p. 166.)

Great, however, as the advantages of the suckling are, they are in some degree counterbalanced by disadvantages which come into operation as soon as the child is dismissed to its nurse in the country. None but the poorest inhabitants of a district offer themselves as wet-nurses, while children who do not require suckling may often be placed with people tolerably well off, sometimes with such as are childless, and who eventually adopt them. M. A. Gendron* states that it is the poorest of all the communes in the arrondissement of Vendôme, that of Percheron, which furnishes the hospice with the greatest number of wet-nurses. Women who have undertaken to suckle a foundling not unfrequently suckle their own child likewise, thus yielding to both an insufficient nourishment; or some will feed the foundling whom they had engaged to suckle, and keep their own child at the breast instead. These are, doubtless, drawbacks from the advantages of attempting to bring up the foundlings at the breast, but they merely show how insufficient are the efforts even of the most active benevolence to do away with all the far-reaching effects of vice.

A paper by M. Villermé in a recent number of the Annales d'Hygiène† contains some elaborate tables illustrative of the influence of suckling and of artificial feeding upon the mortality of foundlings. His facts are derived from the three hospices at Lyons, Rheims, and Paris. In the first, all the children are brought up at the breast; in the second, they are all fed by hand; and in the third, they are usually, though not invariably, suckled by wet-nurses. In the two former hospices the stay of the children is usually limited to one or two days, and never exceeds a week; but they remain in the hospice at Paris for a much longer time.

* Note sur la Création d'un depôt des Enfans Trouvés de Paris, in Annales d'Hygiène, tome vi. p. 81.

† De la Mortalité des Enfans Trouvés, Annales d'Hyg. t. xix. p. 47.

| | Lyons. | Paris. | Rheims. |
|---|---|---|---|
| Mortality under one year, per 100 admissions . . | 33·7 | 50·3 | 63·9 |

The length of the infant's sojourn in the hospice exercises a very considerable influence on their mortality, for many very fatal diseases prevail endemically in those institutions. A third of the children admitted into the hospice at Paris* previous to the French revolution, fell victims to the *endurcissement du tissu cellulaire,* and the disease still continues to carry off great numbers annually. *Muguet* is another very fatal affection, and both it and the induration of the cellular tissue may be regarded as peculiar to foundling hospitals. Pneumonia and gastro-enteritis are likewise very fatal, and the former complicates almost every illness by which the infants are attacked, and greatly diminishes their chances of recovery. To all the above diseases infants are liable in direct proportion to the length of their stay in the hospice; while syphilis, the fear of which has frequently prevented their being placed out at nurse, appears not only to be rarely fatal, but also to occur comparatively seldom. The early reports of the Dublin Foundling Hospital attribute to this disease a very great share in the production of the high mortality, but on an inspection of the children being made in 1799 it was ascertained that only 1 in 29 was so affected.† A similar opinion prevailed in France, but at the time of the general inspection of foundlings in 1821,‡ it appeared that the number of children suffering from venereal affections did not exceed 1 in 17. The importance of making the stay of the foundlings in the hospice as short as possible is now fully appreciated; and M. Remacle (p. 278) mentions that when he visited the hospital at Lyons, its ample ward did not contain above twenty infants.

Even though the interior arrangement of a hospice were perfect, and the stay of the infants there as short as possible, still a new-born child is so frail a being that a thousand precautions are essential to the preservation of its life. The nature of the conveyance by which it is removed from the hospice to its nurse in the country might seem to be a trivial matter; yet to a want of due care in this respect, MM. Terme and Monfalcon attribute a considerable share in producing the mortality among the foundlings belonging to the hospice at Paris. At Lyons messengers are employed, who carry the infants, placed in a cradle and well protected from the weather, on their heads, and this mode of conveying the children is considered preferable to any other, from its exposing the children less to being shaken or injured. We mention this merely as a specimen of the unwearied care with which the conductors of the hospice at Lyons have laboured to attain those favorable results which we must next examine.

There are many other circumstances which peril the life of the foundlings, and it would be interesting if we had room sufficient to trace the steps by which benevolence has sought to combat them, but we must hasten to enquire what have been the fruits of so much care and of such

* Chateauneuf, Lib. cit. p. 80.

Wakefield, Op. cit. vol. ii. chap. xxiv.

‡ Chateauneuf, Op. cit. See the report at the commencement by MM. Duméril and Coquebert-Moubret, p. xix.

lavish expenditure of money. It is true that no marked diminution has yet taken place in the mortality of foundlings throughout France, but the great difference in the rate of that mortality in various departments of the country* would at once suggest that much might be done to reduce it. The attempt has been made in many places, and its first-fruits are just beginning to appear. Nowhere have more zealous or more able workmen engaged in this philanthropic task than at Lyons, and no other city presents such encouraging results. From 1802 to 1810 the total mortality of children under one year compared with the total number at the charge of the hospice, (see Terme and Monfalcon, p. 341 et seq.,) was 1 in 7·4; in the ten succeeding years 1 in 8·65. In 1821 the important improvements in the hospice at Lyons began; and from 1822 to 1831 the mortality, including the still-born children brought to the hospice, was only 1 in 11·3. Since 1831 all the improvements have been brought into full action; all children are brought up at the breast, and their stay in the hospice has been rendered as short as possible. The mortality from 1831 to 1836 has amounted only to 1 in 12·39. Only 1 in 7, or if we exclude the still-born 1 in 15, die before being sent to nurse. In 1820, the mortality during the first year was 50 per cent., but it has since been progressively decreasing, until it now does not exceed 30 per cent. This improvement during the first year of life is observed to continue during the subsequent years; thus, in 1820, the mortality from birth to 12 years was 70 per cent., but had sunk in 1824 to 58·3 per cent., and the expiration of 12 years from 1831 will doubtless show a much greater diminution.

Very many questions connected with this subject remain unnoticed, though interesting alike to the philosopher and the philanthropist. But some conclusion may be drawn from the facts we have already laid before our readers. Foundling hospitals have been the subject of much censure; it has been denied that they have ever done good, and even that they are capable of being turned to good ends. Do they deserve to have such a sentence of condemnation recorded against them? We think not; they are an institution of a by-gone age and an expiring faith. Had they not been what they were they could not have been at all, and we doubt much whether society or morals would have gained by their absence. The motives to charity are changed since the day when the fraternities of the Holy Ghost and St. Lazarus were founded, and with the altered motives there has followed of necessity an altered mode of action. The foundling hospitals in France are undergoing the changes which the times call for, and these changes are being effected by men of great wisdom and benevolence. We think we have shown that their undertaking is by no means a hopeless one; that they have done great good, and are likely to bring about much more; and we would especially recommend the works we have been noticing to all who fancy that there are certain stereotyped forms, according to which charity must needs work, and who deem it impossible that mankind can ever be benefited except by the adoption of their theories and the employment of their remedies.

* Chateauneuf, Op. cit. p. 67.

